# Supplementary material for: Direct true lumen cannulation in type A acute aortic dissection: A review of an 11 years’ experience
Source: PLoS One. 2020 Oct 12;15(10):e0240144. doi: 10.1371/journal.pone.0240144 (PMC7549816; doi:10.1371/journal.pone.0240144)
Supplement: Attachment — Submitted filename: Response to reviewers- DTLC 14.09.2020.docx [file pone.0240144.s002.docx]

**Answers to the reviewers**

We would like to thank you for their positive comments and suggestions. Below, please find our point-by-point answers to the question raised by Reviewer 1 and Reviewer 2. We found their suggestions very helpful and have included their input into our manuscript.

**Comments of Reviewer #1**

528 patients with Type A aortic Dissection (2006-2017) where a unique Direct True Lumen cannulation technique was employed n 52.4% of their patients with similar outcomes (which are acceptable 30-days mortality 12%, 8% new neuro deficits) to their conventional peripheral cannulation techniques (same).

Their novel direct technique involves encircling the dissected aorta (dangerous) and a brief normothermic drop in temperature (also dangerous) and dividing the dissected aorta to place the aortic cannula under direct vision (more dangerous) and would be best reserved for experienced surgeons.

At our center and at Penn, direct cannulation is performed using a “Seldinger” wire technique under epi-aortic ultrasound and TEE guidance, which is much safer (in my opinion) with similar outcomes in obtaining true lumen cannulation and avoiding the pitfalls of peripheral cannulation.

For this article to be published, the discussion should include references to the Seldinger technique and specific reasons why the Mainz center group feels their approach is preferred.
Furthermore, if published the manuscript would benefit from figures showing their preferred technique.

**Responses to Reviewer #1**

1) We believe that antegrade perfusion by central aorta cannulation prevents retrograde cerebral embolization and avoids peripheral arterial injury. As mentioned in sentence number 207-209, cannulation using the Seldinger technique can be time-consuming in emergency cases and may be challenging in the presence of circumferential aortic dissection accompanied with central true lumen collapse.
2) New reference Penn ascending cannulation using the Seldinger technique (ref. no. 23. Schoeneich et al) was added.
3) A video is added to demonstrate the operative technique. Also added is another reference from our department detailing the technique (ref. no. 7. Conzelmann et al).

**Comments of Reviewer #2**

Thanks for your nice report. Another article about femoral cannulation that you might cite is Fusco DS. Ann Thorac Surg 2004 Oct;78(4):1285-9; discussion 1285-9. Your paper could be enhanced by a nice accompanying drawing of your cannulation technique. Although you demonstrated no benefit from this arterial cannulation technique over other methods, the description of your technique is still valuable. How often did you have troubling bleeding from around the cannula during perfusion? Did your surrounding aortic tapes ever tear through the fragile aortic layers. Among the other cannulation options, you mention "left atrial". Please explain.

**Responses to Reviewer #2**

1) The reference (ref. no. 8. Fusco et al) was added as requested.
2) A video is added to demonstrate the operative technique. Also added is another reference from our department detailing the technique (ref. no. 7. Conzelmann et al).
3) In our early experience, after careful dissection between the adventitia of the ascending aorta and the pulmonary artery, we used to place one Mersilene tape around the fragile aorta (ref. no. 7. Conzelmann et al). However, we occasionally encountered some bleeding from around the cannula during CPB perfusion. We now use double Mersilene tape which provide better stability and prevent bleeding from around the aortic cannula.
4) Fortunately, we never had any episode of aortic rupture or tear due to Mersilene tapes in our experience.
5) The left atrial cannulation Reference was added (ref no. 23. Schoeneich et al).
